# A retrospective analysis of honey bee (*Apis mellifera*) pesticide toxicity data

**DOI:** 10.1371/journal.pone.0265962

**Published:** 2022-04-07

**Authors:** Frank T. Farruggia, Kristina Garber, Christine Hartless, Kristin Jones, Lee Kyle, Nicholas Mastrota, Joseph P. Milone, Sujatha Sankula, Keith Sappington, Katherine Stebbins, Thomas Steeger, Holly Summers, Pamela G. Thompson, Michael Wagman

**Affiliations:** Environmental Fate and Effects Division, Office of Pesticide Programs, U.S. Environmental Protection Agency, Washington, DC, United States of America; USDA Agricultural Research Service, UNITED STATES

## Abstract

Current USEPA ecological risk assessments for pesticide registration include a determination of potential risks to bees. Toxicity data are submitted to support these assessments and the USEPA maintains a large database containing acute and chronic toxicity data on adult and larval honey bees (*Apis mellifera*), which USEPA considers a surrogate for *Apis* and non-*Apis* bees. We compared these toxicity data to explore possible trends. This analysis indicated a significant correlation between acute contact and oral median lethal dose (LD_50_) values for adult honey bees (ρ = 0.74, p <0.0001). Using default EPA modeling assumptions, where exposure for an individual bee is roughly 12x lower through contact than through ingestion, the analysis indicates that the oral LD_50_ is similarly if not more protective of the contact LD_50_ for the majority of pesticides and modes of action evaluated. The analysis also provided evidence that compounds with a lower acute toxicity for adults through contact and oral exposure pathways may still be acutely toxic for larvae. The acute toxicity of herbicides and fungicides was higher for larvae relative to oral and contact toxicity for adult honey bees for the same compounds and the no observed adverse effect level (NOAEL) from chronic toxicity studies were lower for larvae relative to adults, indicating increased sensitivity of larvae. When comparing 8-day LD_50_ values between single dose larval acute studies to those derived from repeat dose 22-day larval chronic toxicity studies, the LD_50_ values derived from chronic studies were significantly lower than those from acute toxicity tests (Z = -37, *p* = 0.03).

## Introduction

Insect pollination is required to produce approximately $30 billion worth of agricultural products in the US annually and provides a key ecosystem service within many natural environments [1, 2]. The honey bee (*Apis mellifera*) is frequently used for its pollination services in agriculture [3] and costs associated with pollination services have more than doubled for some crop species since the 1990s [4]. Additionally, considerable losses to both managed and non-managed insect pollinators have called attention to the factors contributing to pollinator declines [5, 6]. Efforts to examine these environmental stressors have led to a consensus in the scientific community that there are multiple factors affecting declines in bee health including parasites (*e*.*g*., *Varroa destructor*), pathogens (multiple viral, bacterial, and fungal diseases), pesticides, lack of adequate nutrition [4], and poor honey bee queen performance [7]. Furthermore, insect pollinators are at risk from habitat loss resulting in declines of floral biodiversity related to conversion of lands to agricultural production and urbanization, bee management practices, lack of genetic diversity and/or changes related to climate [5, 8]. None of these factors have been identified as the single cause of declines; rather, it is the interaction of these factors that appears to be what is most detrimental to bees [8, 9]. Bees can interact with natural and synthetic xenobiotics through a variety of exposure pathways [*e*.*g*., 10] and the role of pesticides as a stressor has been a primary focus of research examining the challenges faced by bees [11].

Organizations charged with determining the potential for ecological risks from pesticides including the USEPA (U.S. Environmental Protection Agency) and the EFSA (European Food Safety Agency) have identified pollinator protection goals and require the submission of pollinator toxicity data when registering pesticides. When examining risks towards pollinators, honey bees are often used as a study species because of their agricultural importance and amenability (*i*.*e*., availability of standardized test method and ready availability) for lab and field-based experimentation [12]. Additionally, honey bees can serve as a surrogate study model for examining risks to other bee species [13]. A multi-tiered approach is used by USEPA when examining pesticide risks to bees during an ecological risk assessment [13].

The *Guidance for Assessing Pesticide Risks to Bees* [13] provides a broad overview of pollinator risk assessment used by the USEPA and outlines the tiered approach used for risk assessment. The initial tier (Tier 1) serves as a screen and consists of laboratory-based acute and chronic toxicity studies with adult and larval honey bees conducted in accord with the Organization for Economic Cooperation and Development (OECD) and/or USEPA Office of Chemical Safety and Pollution Prevention (OCSPP) standardized test guidelines (TG) or guidance documents (GD). Tier 1 toxicity studies include: adult acute contact (AAC; OCSPP 850.3020 [14] and OECD TG 214 [15]); adult acute oral (AAO; OECD TG 213 [16]); the adult chronic oral (ACO; OECD TG 245 [17]), larval acute oral (LAO; OECD TG 237 [18]), and larval chronic oral (LAO; OECD GD 239 [19]). Collectively, these data serve to inform the need for higher-tier colony-level exposure and effect studies intended to provide increased realism at the semi-field (Tier 2; *e*.*g*., OECD GD 75) [20] and full-field (Tier 3; OCSPP 850.3040) [21] level.

Tier 1 studies quantify effects at the individual-level and include measurements for mortality and sublethal effects. During laboratory-based testing, point estimates of acute (*i*.*e*., lethal dose for 50% of the bees tested; LD_50_) and chronic toxicity (*i*.*e*., no-observed adverse effect level; NOAEL) values are calculated for adult and larval worker honey bees for a given pesticide. The toxicity endpoints generated from Tier 1 toxicity studies can then be compared to model estimates of contact/oral exposure to estimate risk to individual bees (as detailed in [13]).

Within the field of toxicology, there is a general interest in reducing, replacing, and refining the current reliance on animal testing [22, 23]. While these efforts have historically focused on vertebrates, new approach methodologies such as high-throughput *in vitro* assays and predictive models (computational toxicology) are emerging that leverage existing data and show promise toward more efficient yet equally protective means of evaluating risk across a broader range of taxa (*i*.*e*., vertebrate and invertebrate). While reduced reliance on whole animal testing is an important benefit of such efforts, the development of predictive models and *in vitro* assays or possible reductions in data requirements must be based on sound science demonstrating that such tools are relevant and reliable or that other lines of evidence are suitably predictive if they are to gain regulatory acceptance [24]. Over the last several years USEPA has received hundreds of Tier 1 honey bee studies through the pesticide registration and reregistration processes under the Federal Insecticide, Fungicide, and Rodenticide Act (FIFRA) (7 U.S.C. §136 *et seq*.). This analysis is intended to examine patterns among these data and assess whether efficiencies can be gained in targeting the specific studies needed for risk assessment.

This analysis utilizes EPA’s honey bee toxicity database to compare the relative toxicity of insecticides, fungicides and herbicides across acute (single dose) and chronic (repeat dose) tests with adult and larval worker bees. The objective of this analysis was to explore the relative sensitivity of larval bees versus adults and to determine the predictive utility of: acute contact toxicity data relative to acute oral toxicity in adults; of acute toxicity data for chronic toxicity; and of chronic (repeat dose) larval toxicity data for acute (single dose) larval toxicity.

## Methods

This retrospective analysis includes toxicity data from Tier I honey bee toxicity studies conducted in accordance with OECD and/or OCSPP test guidelines/guidance documents including: the adult acute contact (AAC; OCSPP 850.3020 [14] and OECD TG 214 [15]); adult acute oral (AAO; OECD TG 213 [16]); the adult chronic oral (ACO; OECD TG 245 [17]), larval acute oral (LAO; OECD TG 237 [18]), and larval chronic oral (LCO; OECD GD 239 [19]) honey bee toxicity studies. While the larval toxicity tests focus primarily on the ingestion of pesticide residues in diet, exposure in these studies is not limited to oral ingestion but includes contact exposure as well. Also, while the chronic toxicity studies with larval bees typically report exposure in terms of cumulative dose (*i*.*e*., μg ai/larva), USEPA calculates exposure from these studies in terms of daily dose (*i*.*e*., μg ai/larva/day). USEPA recognizes that it is not possible to readily determine the actual volume of diet consumed by the developing larvae each day the organism is dosed. Therefore, daily dose is calculated by dividing the cumulative dose by 4 (*i*.*e*., the number of days over which the larvae are dosed).

### Pesticide classifications

#### Chemical classes and modes of action (MOA)

For some comparisons, we organized pesticides into categories by chemical class (*e*.*g*., organophosphates), pesticide class (*e*.*g*., insecticides), and chemical mode of action (MOA) (S1 Table). When comparing across adult acute oral and contact toxicity data, we categorized pesticides into classes (*e*.*g*., fungicide, herbicide, insecticide) using multiple reference databases (See pesticide classification sources in S2 Table). Additionally, we further sub-classified insecticides using general MOAs established by the Insecticide Resistance Action Committee (IRAC; https://irac-online.org/) and the Pesticide Properties Database (See S3 Table). For all other comparisons, we categorized pesticides using a general MOA, and a brief description MOA [25] to further characterize each compound (See sources of information in S2 Table). Pesticide classifications and MOAs are summarized in S1 Table.

#### Toxicity classifications

When comparing the relative acute oral toxicity across pesticides for both adults and larvae, we relied upon the following USEPA acute toxicity classification categories [26]:

- Practically Non-toxic (LD_50_ ≥ 11 μg a.i./bee);
- Moderately Toxic (2 μg a.i.< LD_50_ < 11 μg a.i./bee); and,
- Highly Toxic (LD_50_ ≤ 2 μg a.i./bee).

Non-definitive toxicity endpoints include values which are greater than (>) the highest tested dose or are less than (<) the lowest dose level. When classifying toxicity values, we categorized non-definitive endpoints, which had “>” values as if they were definitive. Because of their non-definitive nature, we distinguished these values from the definitive endpoints when representing them visually. If an endpoint value was < 2 μg a.i/bee, we classified it as “highly toxic non-definitive.” If an endpoint had either < or > signs expressed within the range of LD_50_ = 2–11 μg a.i./bee, we classified it as “Moderately Toxic non-definitive.” Lastly, if a compound had a non-definitive LD_50_ >11 μg a.i./bee, we classified it as “Practically Non-toxic” with no distinction between definitive and non-definitive.

### Source of endpoint data

For data curation and screening, within the USEPA Office of Pesticide Programs (OPP), the Environmental Fate and Effects Division (EFED) routinely produces Data Evaluation Records (DERs), which represent the agency’s independent review, evaluation, and endpoint selection for registrant-submitted (unpublished) and open literature pesticide toxicity studies. The toxicity endpoints and primary study information from the honey bee toxicity studies are available in though EPA’s ECOTOXicology Knowledgebase (https://cfpub.epa.gov/ecotox/). All mortality-based honey bee toxicity endpoints included in this retrospective analysis are extracted from DERs that were completed prior to August 2019. Due to this cut-off date, some chemicals and endpoints were not included in the analysis. During USEPA’s review process, each DER is assigned a specific study classification based on quality and adherence to established guidelines. Studies classified as “acceptable” fully adhere to the standardized testing guidelines while “supplemental” studies deviate somewhat from the standardized testing conditions, but the deviations are not considered serious enough to compromise scientific integrity of the results. In this analysis, we only included studies and their endpoints if they were classified as either “acceptable” or “supplemental.” Studies classified as “invalid” are not scientifically sound and were excluded from further analysis. Additionally, we only included endpoints from studies testing technical grade active ingredients (TGAI; active ingredient alone); therefore, data on typical end-use products (TEP; active and inert ingredients) were not included in the analysis. See S4 Table for a complete listing of studies that were considered after applying the screening criteria.

### Study and endpoint selection

When performing an analysis of adult bee acute oral and contact toxicity we compared the lethal dose to 50% of the organisms tested (*i*.*e*., LD_50_) from AAO and AAC studies. We excluded pesticides not currently registered for use in the U.S. and toxicity endpoints reported in non-standard units that couldn’t be converted to μg a.i./bee. Available endpoints from these acute adult studies are often non-definitive (*i*.*e*., the LD_50_ > maximum tested concentration) because of maximum dose requirements for testing under the guidelines. Despite the uncertainty associated with non-definitive endpoints, they can provide meaningful information for risk assessment [27] and their exclusion would have limited the dataset to compounds with a relatively high acute toxicity towards honey bees (*e*.*g*., insecticides). The analysis included non-definitive values in comparisons where there was one definitive value and one non-definitive value (no comparisons were made between two non-definitive endpoints). This analysis also utilized larval LD_50_ values generated from LAO and LCO study guidelines. For pesticides with multiple toxicity endpoints derived from independent studies, the analysis utilized the lowest or most definitive LD_50_ value. For example, if two studies resulted in LD_50_ values of 0.5 and 0.1 μg a.i./bee, we used 0.1 μg a.i./bee as the LD_50_ value. When both non-definitive values and definitive value(s) were present, the analysis utilized the definitive value. The analysis also excluded acute toxicity data extending beyond the 48-hour time interval to ensure the endpoints represented the same study duration. S1 Table lists all included acute adult endpoints.

When comparing oral acute and chronic toxicities, we included mortality data from honey bee adult and larval toxicity studies that adhered to four OECD test guidelines: AAO; ACO; LAO and LCO. This included the estimated LD_50_ (μg a.i./bee or μg a.i./larva) from both adult and larval acute (single dose) toxicity study guidelines (AAO and LAO) as well as the Day 8 (D8) LD_50_ estimates from larval chronic (repeat dose) toxicity studies (LCO). Endpoints from chronic studies can be expressed as the cumulative dose consumed during the exposure period (μg a.i./bee) or as the daily dose consumed per individual (μg a.i./bee/day). We compared all endpoints in common units (*i*.*e*. μg a.i./bee/day or μg a.i./larva/day) because daily dose is used by the current USEPA exposure model for honey bees during ecological risk assessments [13] and is the basis for understanding relative toxicity at USEPA. EPA relies upon daily dose-based exposures for both acute and chronic based exposures because it is important to review the comparable relationships between acute and chronic endpoints using the same units. This approach is utilized because risk to developing honey bee larvae is assessed based on daily exposures via food consumption not cumulative food consumption during the entire larval stage.

We excluded endpoints based on dietary concentrations such as median lethal concentration (LC_50_) and no/lowest observed adverse effect concentration (NOAEC and LOAEC; *i*.*e*. mg a.i./kg diet). Although typically reported, we did not use NOAEL and LOAEL values from the acute studies (AAO and LAO) in any analysis. Additionally, we excluded any studies on chemical degradates, studies containing multiple active ingredients, and TEP-based exposures from the analysis. As noted earlier, while ingestion of treated diet (*i*.*e*., oral exposure) is the primary focus of the larval toxicity studies, it is important to note that larvae are also in direct contact during the bioassay. However, measurement endpoints from the larval studies are evaluated in risk assessment assuming the oral route of exposure is dominant [18, 19].

### Adult oral and contact endpoint sensitivity ratio

When comparing the relative toxicities between oral and contact endpoints for a given pesticide, we employed a relative sensitivity ratio (*R*), using LD_50_ values standardized by μg a.i./bee.


$$R=\frac{\text{Acute}\;\text{adult}\;\text{oral}\;{\text{LD}}_{50}\;(\mu \text{g}\;\text{a}.\text{i}./\text{bee}/\text{day})}{\text{Acute}\;\text{adult}\;\text{contact}\;{\text{LD}}_{50}\;(\mu \text{g}\;\text{a}.\text{i}./\text{bee}/\text{day})}$$

A value of 1 signifies no difference between oral and contact toxicity; whereas values less than 1 represents higher oral toxicity and values greater than 1 represent higher contact toxicity. The absence of sample variance data corresponding to the reported LD_50_ values precluded statistical comparisons for individual ratio differences. Instead, we relied on nominal thresholds at five and ten-fold differences above and below the 1:1 relationship benchmark to evaluate the strength of any identified relationships. We required at least one definitive endpoint to perform the analysis. All non-definitive endpoints included in this comparison were greater than (>) values. Ratios calculated using both a non-definitive and a definitive endpoint provide a conservative estimate because a non-definitive (*i*.*e*., greater than) endpoint is an underestimation of the actual LD_50_ and is typically derived from the highest tested dose in an individual study as the actual LD_50_ is higher than what was actually tested.

### Adult and larval chronic and acute comparisons

#### Acute-to-chronic toxicity ratio (ACR) calculation

Acute-to-chronic toxicity ratios (ACR) serve as a means to estimate the acute or chronic toxicity of a chemical towards an organism when only acute or chronic toxicity data are available [28]. We calculated ACRs for honey bee mortality endpoints as the ratio of the LD_50_ value from the acute oral toxicity studies (either AAO or LAO) to the most sensitive mortality NOAEL from the chronic toxicity study (either ACO or LCO):

$$ACR=\frac{Median\;Lethal\;Dose\;({LD}_{50})}{No\;Observed\;Adverse\;Effect\;Level\;(NOAEL)}$$

The ACO (adult) studies provide only a single mortality NOAEL endpoint; however, the LCO (larval) studies, multiple mortality NOAEL values (*i*.*e*., larval mortality at Day 8; pupal mortality at Day 15; and adult mortality or emergence at Day 22). In this analysis, we focused on the most sensitive mortality NOAEL endpoint when calculating larval ACRs. Additional measurement endpoints (*e*.*g*., food consumption; adult bee weight) are also provided in these studies and occasionally may represent a more sensitive response than mortality. However, to minimize potential confounding factors, these endpoints are not included in this comparison.

### Comparison of larval LD_50_ estimates derived from acute and chronic studies

Larval chronic oral toxicity studies (LCO) are primarily aimed at determining NOAEL and LOAEL values; however, an LD_50_ value on Day 8 can also be calculated. Despite being able to derive the same endpoint as larval acute (single dose) oral toxicity studies, LCO studies use a repeat daily dosing (typically four consecutive days) while LAO studies evaluate the effects of a single dose. USEPA recognizes that the likelihood of acute (single dose) exposure for larval honey bees may be limited under natural conditions; however, the LD_50_ value is useful in characterizing the acute toxicity of a pesticide and is consistent with what is done for other taxa (*e*.*g*., birds, mammals, fish, aquatic invertebrates). When both acute and chronic larval toxicity studies were available for a chemical, we compared the single dose larval acute LD_50_ from the LAO study to the day eight (D8) LD_50_ estimates from the LCO study. If multiple LD_50_ values were available for a single active ingredient under either study guideline, we selected the most sensitive LD_50_ for comparisons. The analysis excludes non-definitive LD_50_ values. We compared endpoints using a ratio of the LAO to LCO LD_50_ values:

$$\frac{LAO\;based\;Larval\;{LD}_{50}\;(\mu g\;a.i./larva/day)}{LCO\;based\;Larval\;{LD}_{50}\;(\mu g\;a.i.\;/\;larva/day)\;}$$

### Statistical analysis, data visualization, and risk comparisons

The analysis utilized Microsoft Excel to perform calculations and visualizations for sensitivity comparison ratios across study types and pesticide classifications. We used linear regression and a Spearman’s correlation analysis when investigating the relationship between log-transformed definitive AAO and AAC LD_50_ endpoints in JMP^®^ Pro 14 (SAS Institute inc., Cary, NC). We produced additional visualizations using JMP^®^ Pro 14. Lastly, a Wilcoxon Signed Rank Test on matched pairs (α = 0.05) was used to compare the LAO and LCO based LD_50_ estimates. When comparing experimental endpoints to estimated exposures through food consumption and contact for adults and larvae, we relied upon default exposure factors included in BeeREX v.1.0 (https://www.epa.gov/pesticide-science-and-assessing-pesticide-risks/models-pesticide-risk-assessment#beerex) and as described in the USEPA 2014 [13].

## Results

In total, 1,888 studies in the database were considered for analysis prior to data screening. Then screening criteria were implemented to ensure only the most robust comparisons were generated (see Methods). As a result of the screening criteria, the analysis excluded 60% of the total dataset. For the studies that passed the initial screen, the adult acute contact toxicity studies (AAC; [14, 15]) represented the greatest percentage (412; 52%) of the total followed by the adult acute oral toxicity studies (AAO; [16]) (136; 18%). The other three toxicity studies adult chronic oral (ACO; [17]), larval acute oral (LAO; [18]) and larval chronic oral (LCO; [19]), comprised a similar and much smaller proportion of the included studies with 9% (76), 7% (58) and 13% (106) respectively. After the most sensitive endpoints were identified from each study, and duplicate studies for each chemical were removed, a total of 195 chemicals, representing 115 chemical classes and 24 general mechanisms of action, had at least one study representing two or more of the test guidelines (S1 Table).

While there were often more chemicals with honey bee studies available, comparisons were limited based upon the study pairs within each chemical and the nature of the endpoint data (i.e., definitive vs non-definitive; Table 1). For example, following data curation and screening, we identified 146 chemicals which had both acute oral and contact toxicity values for adult bees (S1 Table), but a large portion of these had non-definitive values for both studies and therefore were excluded from analyses. Of these chemicals, fungicides and herbicides had a high number of chemicals with both studies being non-definitive toxicity values (*i*.*e*., 33 of 40 fungicides and 50 of 60 herbicides; S1 Table). Whereas only 10 of 41 insecticides had non-definitive LD_50_ values for both studies. Based on the requirement of at least one endpoint being definitive, there were 49 chemicals that had endpoints that could be compared. The final dataset for comparing the results of oral and contact studies for the same pesticide, was therefore limited to 31 insecticides, 10 herbicides, 7 fungicides and one nematicide (S1 Table).

**Table 1:** Summary of the number of studies and chemicals within each study guideline tested as technical grade active ingredient (TGAI) that were considered before and after data screening.

Target Chemistry	Adult acute contact (AAC; 850.3020/OECD TG 214)	Adult acute oral (AAO; OECD TG 213)	Adult chronic oral (ACO; OECD TG 245)	Larval acute oral (LAO; OECD TG 237)	Larval chronic oral (LCO; OECD GD 239)
Number of studies in database^1^	1222	347	112	75	132
Number of Chemicals with each study^1^^,^ ^2^	167 (41)	152 (40)	69 (69)	58 (34)	96 (32; 95)^4^
Herbicides^1^^,^^2^	69 (8)	62 (7)	32 (32)	26 (14)	46 (13; 45)
Fungicides^1^^,^^2^	46 (3)	43 (5)	16 (16)	15 (5)	30 (8; 30)
Insecticides^1^^,^^2^	46 (29)	42 (28)	19 (19)	16 (14)	19 (11; 19)
Other^2^^,^ ^3^	6 (1)	5 (0)	2 (2)	1 (1)	1 (0; 1)

^1^ These numbers represent total available studies, however not all studies could be used in all comparative analyses.

^2^ Numbers in parentheses represent the number of chemicals with definitive adult acute LD_50_ endpoints. Chronic and larval toxicity endpoitns were all definitive.

^3^ Other includes: nematicides, molluscicides, miticides, and microbicides.

^4^ LCO studies provide both LC50 and NOAEL estimates, the numbers in parenthesis reflect the number of definitive endpoints for each respectively.

The primary toxicity endpoint from the adult (AAO) and larval (LAO) acute oral toxicity studies is the LD_50_; whereas, the chronic studies (ACO and LCO) are used to calculate multiple endpoints. In the available dataset, AAO studies had a relatively low proportion (31%) of definitive toxicity endpoints; however, NOAEL values from ACO tests were all definitive (Table 1). For the larval toxicity studies, most of the NOAEL endpoints included in the retrospective were set at the highest tested concentration because no-effects were detected (*i*.*e*., no LOAELs); however, 65% of the larval LD_50_ endpoints were definitive (S1 Table). The analyses between adult and larval chronic and acute toxicity studies utilized only the definitive endpoints.

### Relative toxicity patterns

**Fig 1:**
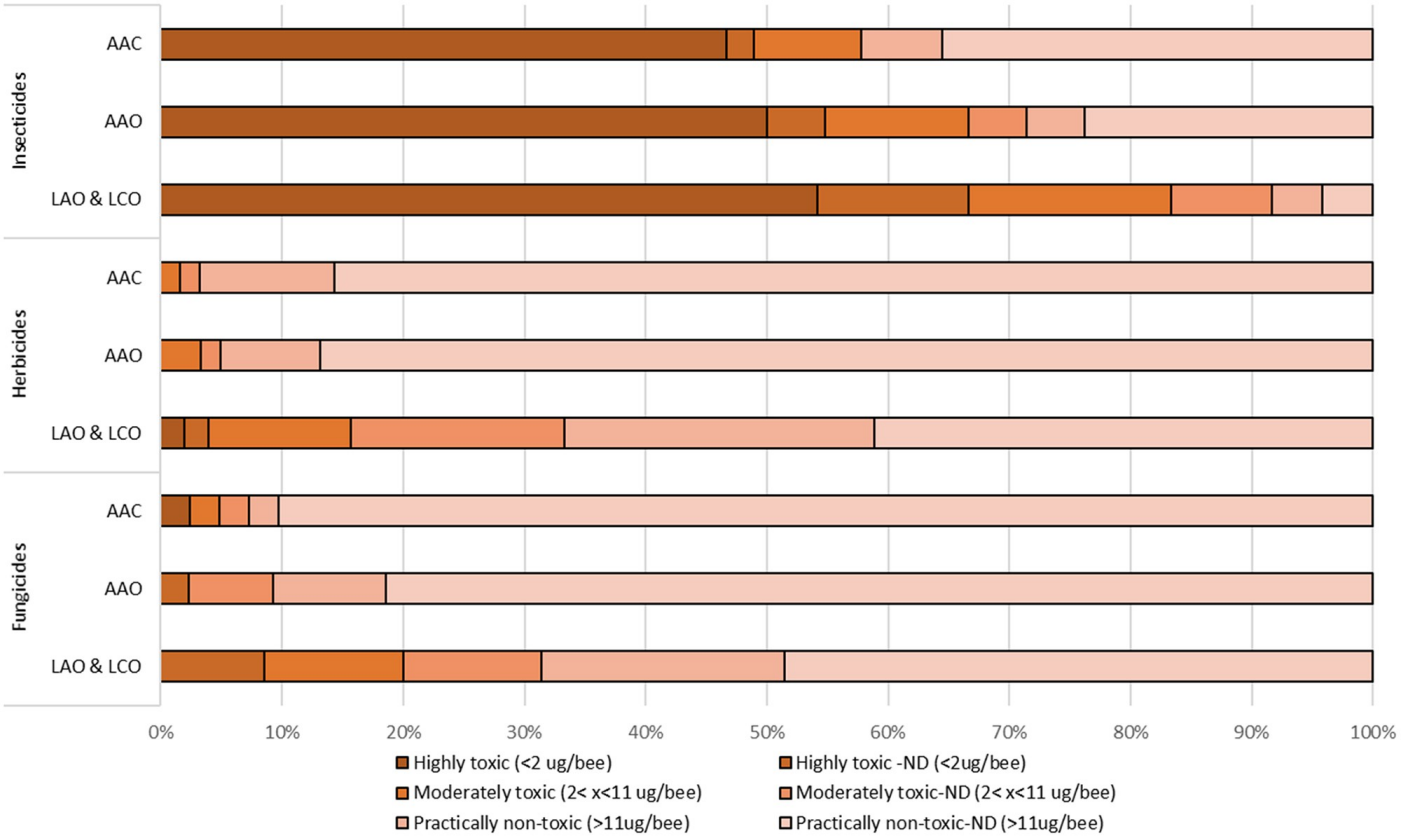
Proportional relationships of toxicity categories across adult and larval life stages and exposure based on contact and oral LD_50_ values for insecticides, herbicides, and fungicides. AAC- Adult Acute Contact (n = 149), AAO- Adult Acute Oral (n = 146), LAO Larval Acute Oral & LCO—Larval Chronic Oral LD_50_s (n = 110).

All available LD_50_ data, provided in S1 Table, were classified as either highly toxic (LD_50_ ≤ 2 μg/bee), moderately toxic (2 < LD_50_ < 11 μg/bee) or practically non-toxic (LD_50_ >11 μg/bee) on an acute exposure basis. Fig 1 illustrates that based on the adult acute LD_50_, insecticides are primarily classified as highly- or moderately toxic on an acute exposure basis; however, herbicides and fungicides are predominately classified as practically non-toxic to bees on an acute exposure basis. Non-definitive (ND) endpoints for highly and moderately toxic categories are provided separately; these are combined with definitive endpoints for the practically non-toxic category (defined as LD_50_ ≥11ug/bee). Larval LD_50_ values generated from LAO and LCO study guidelines are combined, the most sensitive estimate was plotted for chemicals that had estimates for both. For the larval LD_50_ values the patterns are similar; however, a greater proportion of herbicides and fungicides were classified as highly to moderately toxic.

#### Comparison of AAC and AAO LD_50s_

**Fig 2:**
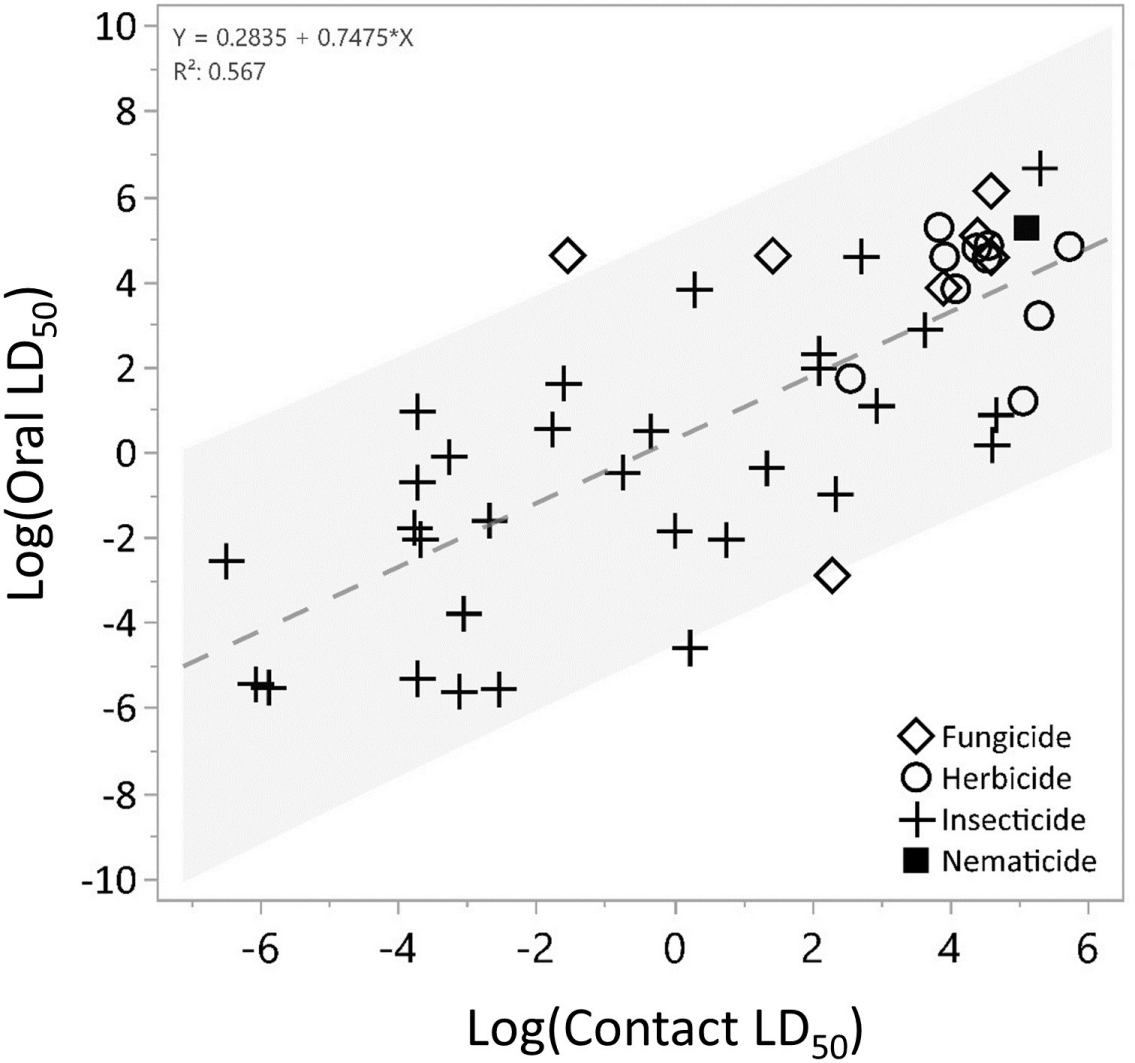
Linear regression between log-transformed adult acute oral and acute contact LD_50_ values (Spearman’s ρ = 0.74, p <0.0001, n = 49 study pairs) by chemical. The shaded region illustrates the predicted 95% confidence interval.

Overall, there was a significant positive correlation between AAO and AAC log transformed endpoints (Spearman’s ρ = 0.74, p <0.0001, n = 49 study pairs, Fig 2). For the included chemicals, 83% had less than a five-fold difference between the two endpoints, while 86% had less than a ten-fold difference.

**Fig 3:**
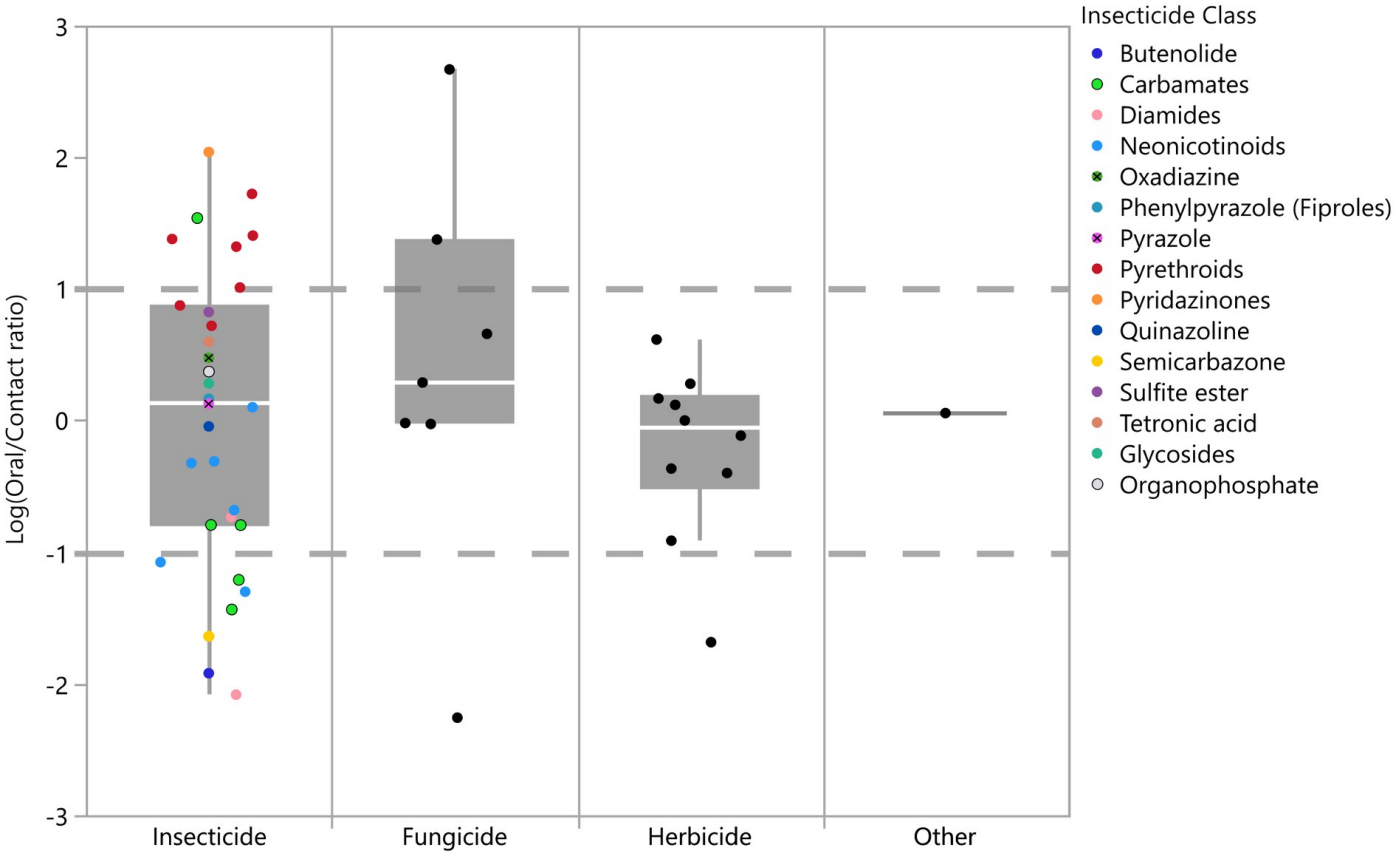
Ratios of adult honey bee (*Apis mellifera*) acute oral and contact LD_50_ value across pesticide classes. Presented data are log_10_ (ratio of oral LD_50_ to contact LD_50_). Dashed lines represent ten-fold deviations from a 1:1 relationship.

When comparing ratios (“*R values”*) between oral and contact LD_50_ values there is a generalized pattern that suggests the acute oral LD_50_ is as sensitive if not more sensitive than the acute contact LD_50_, as illustrated in Fig 3 when log_10_ *R* ≤ 0. Nine pesticides, 7 neurotoxicant insecticides (*i*.*e*., clothianidin, flupyradifurone, imidacloprid, metaflumizone, oxamyl, tetraniliprole, and thiodicarb), one fungicide (captan) and 1 herbicide (bromoxynil), had *R values* ≤ 0.1 (R = 0.08, 0.012, 0.05, 0.02, 0.04, 0.008, 0.06, 0.005, and 0.02, respectively) and are depicted in Fig 3 when log_10_ *R* ≤ -1 which signifies a higher degree of oral toxicity (10-fold or more) relative to contact toxicity. Comparatively, eight pesticides had *R values* ≥ 1, indicating relatively higher acute contact toxicity for these pairs (10-fold or more). These nine pesticides included 6 neurotoxicant insecticides (*i*.*e*., tefluthrin, transfluthrin, *lambda*-cyhalothrin, momfluorothrin, carbaryl and deltamethrin; *R* = 10, 21, 24, 25, 34 and 53 respectively), one respiration inhibitor/regulator insecticide (*i*.*e*., pyridaben; *R* = 110), and two biosynthesis inhibiting fungicides (*i*.*e*., fenhexamid and spiroxamine; *R* = 465 and 24 respectively). These are depicted in Fig 3 as log_10_ *R values* ≥ 1.

The USEPA’s pesticide risk assessment process for bees assumes that the estimated environmental concentrations (EECs) for adult contact and oral routes of exposure are approximately 12x different (*i*.*e*., oral EEC > contact EEC; see [13]). This is because pesticide exposure though ingestion of residues in diet is expected to occur at a higher rate (12x) than would occur from contact exposure (absorption through the integument); therefore, the contact toxicity of a chemical would need to be 12X more toxic to represent an equivalent level of risk. Sixteen percent (8/49) of the chemicals had adult acute contact LD_50_ values 12x or more toxic than the adult acute oral LD_50_. These exceptions included 6 contact insecticides (*i*.*e*., pyridaben, deltamethrin, momfluorothrin, *lambda*-cyhalothrin, carbaryl, and transfluthrin) and 2 fungicides (*i*.*e*., fenhexamid, spiroxamine) with non-definitive oral LD_50_ values.

#### Comparison of LAO and LCO based LD_50s_

Comparisons were made (Table 2) between the LD_50_ values from the larval acute (single dose) oral toxicity studies (LAO; OECD TG 237) and the Day 8 LD_50_ estimates from the larval chronic (repeat dose) studies (LCO; OECD GD 239). The LD_50_ values from the LAO studies and those derived from LCO studies were found to be significantly different (Wilcoxon Signed Rank Test on matched pairs, n = 15 Z = -37, *p* = 0.03) with LD_50_ values derived from chronic tests being significantly lower than those from single exposure larval testing. The LD_50_ comparisons were made directly between 15 individual pesticide active ingredients derived from 15 different chemical classes spanning 6 different MOAs with several studies representing neurotoxicants and biosynthesis inhibitors. The comparison between the LAO and LCO resulted in only two chemicals (*i*.*e*., acetamiprid and MCPA) where the single dose LD_50_ value was more sensitive than the repeat dose LD_50_ value.

**Table 2:** Comparison of larval honey bee (*Apis mellifera*) acute (LAO; single dose; OECD TG 237) and chronic (LCO; repeat dose; OECD GD 239) LD_50_ values (N = 15) expressed in terms of μg ai larva^-1^ day^-1^.

Pesticide	Type	Chemical Class	General MOA	LAO LD_50_	LCO LD_50_	Ratio Single: Repeat dose LD_50_
Ipconazole	Fungicide	Triazoles	Biosynthesis Inhib.	22	14	1.6
MCPA	Herbicide	Chlorophenoxy acid/ ester	Growth Regulator	34.6	93.9	0.4
Cycloate	Herbicide	Thiocarbamate	Biosynthesis Inhib.	10	8.4	1.2
Prosulfuron	Herbicide	Sulfonylureas	Biosynthesis Inhib.	26	21	1.2
Acetochlor	Herbicide	Chloroacetanilide	Biosynthesis Inhib.	26.5	11.8	2.2
Forchlorfenuron	Herbicide	Phenylureas	Growth Regulator	15	4.23	3.5
Pinoxaden	Herbicide	Phenylpyrazoline	Biosynthesis Inhib.	5.2	1.4	3.7
Amicarbazone	Herbicide	Triazolinones	Photosynthesis	45	5.2	8.7
Acetamiprid	Insecticide	Neonicotinoids	Neurotoxicant	1.16	5.43	0.2
Abamectin	Insecticide	Glycosides	Neurotoxicant	0.0011	0.0005	2.2
Tetraniliprole	Insecticide	Diamide	Neurotoxicant	0.013	0.0046	2.8
Tolfenpyrad	Insecticide	Pyrazole	Respiration Inhib./Reg.	0.044	0.014	3.1
Propargite	Insecticide	Sulfite ester	Respiration Inhib./Reg.	25.31	4.8	5.3
Fenpyroximate	Insecticide	Phenoxypyrazoles	Respiration Inhib./Reg.	0.2	0.029	6.9
Sulfoxaflor	Insecticide	Sulfoximine	Neurotoxicant	2.65	0.0494	53.6

#### Acute toxicity endpoints: Adult (AAO) and larval (LAO, LCO) LD_50_ values

The acute toxicity of various pesticides to adult and larval honey bees were compared using the LD_50_ values determined in their respective studies. The AAO toxicity data were available for 152 pesticides. Since the LAO studies were more limited in number than any other guideline (Table 1), the LCO studies were also relied upon for estimating the larval LD_50_ values which expanded the total number of pesticides to 112 (Table 3). When both LAO and LCO values were available, and only one was definitive, the definitive endpoint was selected. If both LAO and LCO endpoints were available and were definitive, the lower (more sensitive) LD_50_ value was selected. Table 3 provides the distribution of the definitive and non-definitive LD_50_ data for adults and larvae across the types of pesticides. The non-definitive data were provided here to illustrate that very few fungicides and herbicides had definitive values from the AAO test.

**Table 3:** Distribution of all available acute toxicity data (LD_50_ values) for honey bee (*Apis mellifera*) adults and larvae. AAO- Adult Acute Oral, LAO- Larval Acute Oral, LCO- Larval Chronic Oral.

Endpoint Characteristics and Pesticide Class		Adult (AAO) LD50s				Larval (LAO and LCO) LD50s			
		Fungicides	Herbicides	Insecticides	Other^1^	Fungicides	Herbicides	Insecticides	Other^1^
Definitive	n	4	7	28	0	11	20	18	1
	min	47	3.3	0.004		4.8	1.4	0.0005	-
	max	455	127	792.4		60	87.2	63	72
	median	91.8	45.9	0.3		14.8	20.5	0.6	-
Non-definitive	n	39	54	14	5	24	31	6	1
	min	>0.9	>11	>0.1	>0.4	>0.004	>1.1	>0.005	-
	max	>13,443	>445	>200	>200	>150	>100	>14.5	>1
Total	n	43	61	42	5	35	51	24	2
Proportion definitive		0.09	0.11	0.67	0.00	0.31	0.39	0.75	0.50

^1^ “Other” group includes nematicides and miticides.

#### Direct chemical comparisons across adult and larval LD_50_ values

Only 11 pesticides had both adult and larval acute oral LD_50_ values which were definitive (Table 4). The adult LD_50_ was compared to either or both of the larval acute LD_50_ estimates from the LAO or LCO (8-day LD_50_s) studies. The 11 pesticides span 10 different chemical classes, but only include insecticides and one herbicide, and eight of the insecticides are neurotoxicants. Notably, this dataset only includes one neonicotinoid, due to the cut off in timing for DERs and endpoints that were available for this analysis. For each of the comparisons (LAO: AAO or LCO:AAO) 8 pesticides could be compared. The AAO LD_50_ value was more sensitive than the LAO and LCO based LD_50_ value in 5 and 1 of the pesticides respectively (Table 4).

**Table 4:** Comparison of honey bee (*Apis mellifera*) adult acute (AAO; OECD TG 213), larval acute (LAO; OECD TG 237), and Larval Chronic Oral (LCO; OECD GD 239) technical grade active grade ingredients (TGAI) LD_50_ values (N = 11). Units for the adult and larval LD_50_ values are μg ai bee^-1^ and μg ai larva^-1^, respectively.

Pesticide	General Class	Chemical Class	General MOA	Adult AAO LD_50_	Larval LAO LD_50_	Larval LCO Day 8 LD_50_	LAO:AAO LD_50_ Ratio	LCO:AAO LD_50_ Ratio
Fenazaquin	Insecticide	Quinazoline	Respiration Inhib./Reg.	5.8	0.347	-	0.06	-
Tolfenpyrad	Insecticide	Pyrazole	Respiration Inhib./Reg.	0.63	0.044	0.014	0.07	0.02
Abamectin	Insecticide	Glycosides	Neurotoxicant	0.0044	0.0011	0.000498	0.25	0.11
Cyclaniliprole	Insecticide	Diamide	Neurotoxicant	0.702	-	0.05225	-	0.07
Tetraniliprole	Insecticide	Diamide	Neurotoxicant	0.0103	0.013	0.0046	1.26	0.45
Amicarbazone	Herbicide	Triazolinones	Photosynthesis	24.4	45	5.2	1.84	0.21
Oxamyl	Insecticide	Carbamate	Neurotoxicant	0.379	0.931	-	2.46	-
Fipronil	Insecticide	Phenylpyrazoles	Neurotoxicant	0.00405	0.0218	-	5.38	-
Sulfoxaflor	Insecticide	Sulfoximine	Neurotoxicant	0.146	2.65	0.0494	18.15	0.34
Formetanate hydrochloride	Insecticide	Carbamate	Neurotoxicant	0.16	-	0.11	-	0.69
Thiamethoxam	Insecticide	Neonicotinoids	Neurotoxicant	0.005	-	0.78	-	156

#### Comparison of mortality-based NOAEL endpoints from ACO and LCO studies

**Fig 4:**
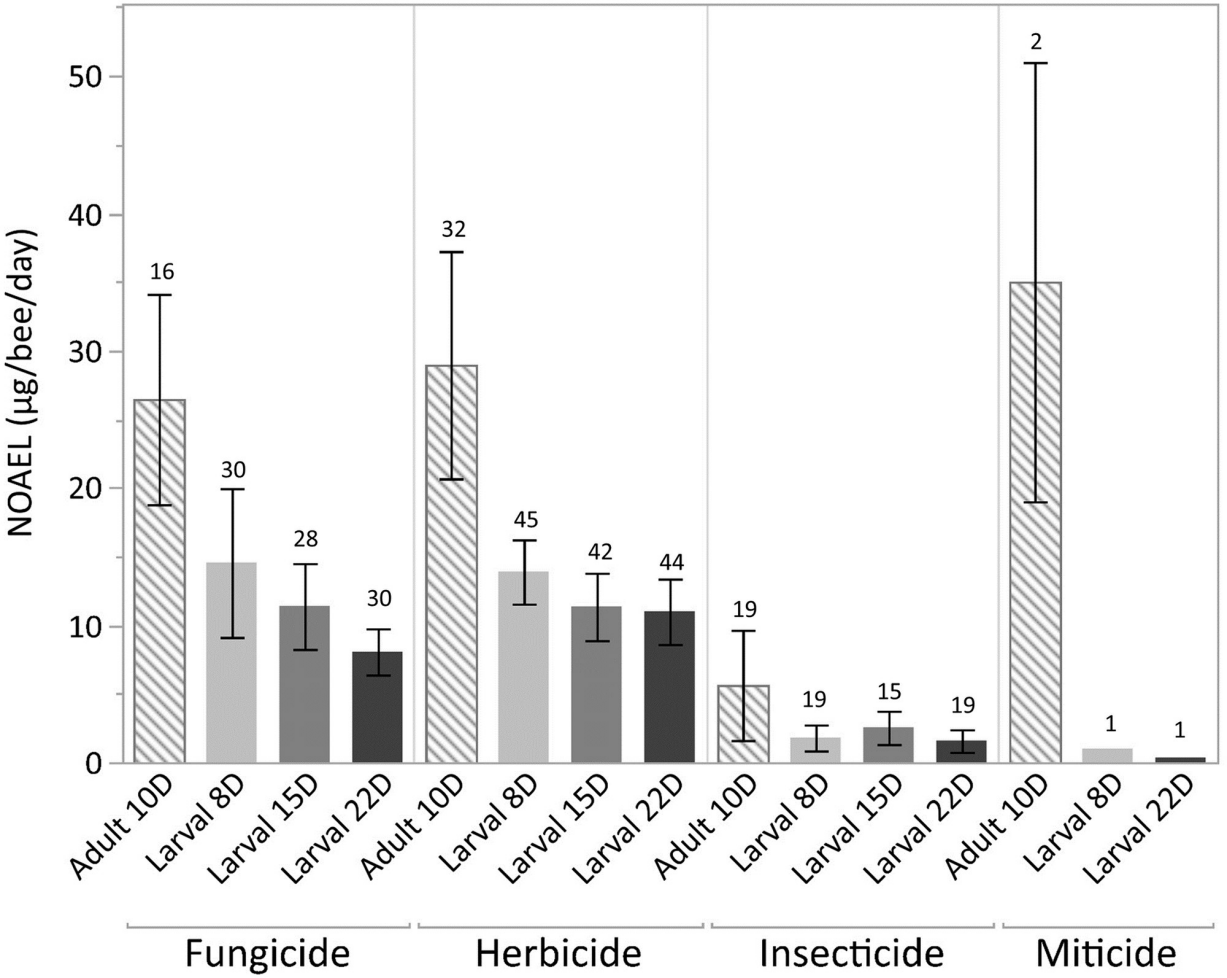
Average no-observed adverse effect levels (NOAEL; bars are mean standard errors) for honey bee (*Apis mellifera*) chronic adult (ACO; OECD GD 245) and chronic larval toxicity studies (LCO; OECD GD 239). Sample sizes are displayed above each bar.

The available dataset contains a total of 96 LCO studies, most of which (85%) reported a mortality-based NOAEL and LOAEL from at least one of the time points (*i*.*e*., larval Day-8, pupal Day-15 or adult Day-22). There were 69 ACO toxicity studies, with 59% of them having both mortality-based NOAEL and LOAEL values. Fig 4 illustrates the general pattern of increased toxicity (*i*.*e*., more sensitive NOAEL values) of fungicides, herbicides and miticides to larvae relative to adults, while for insecticides the average NOAEL for adults and larvae was within one standard error.

**Fig 5:**
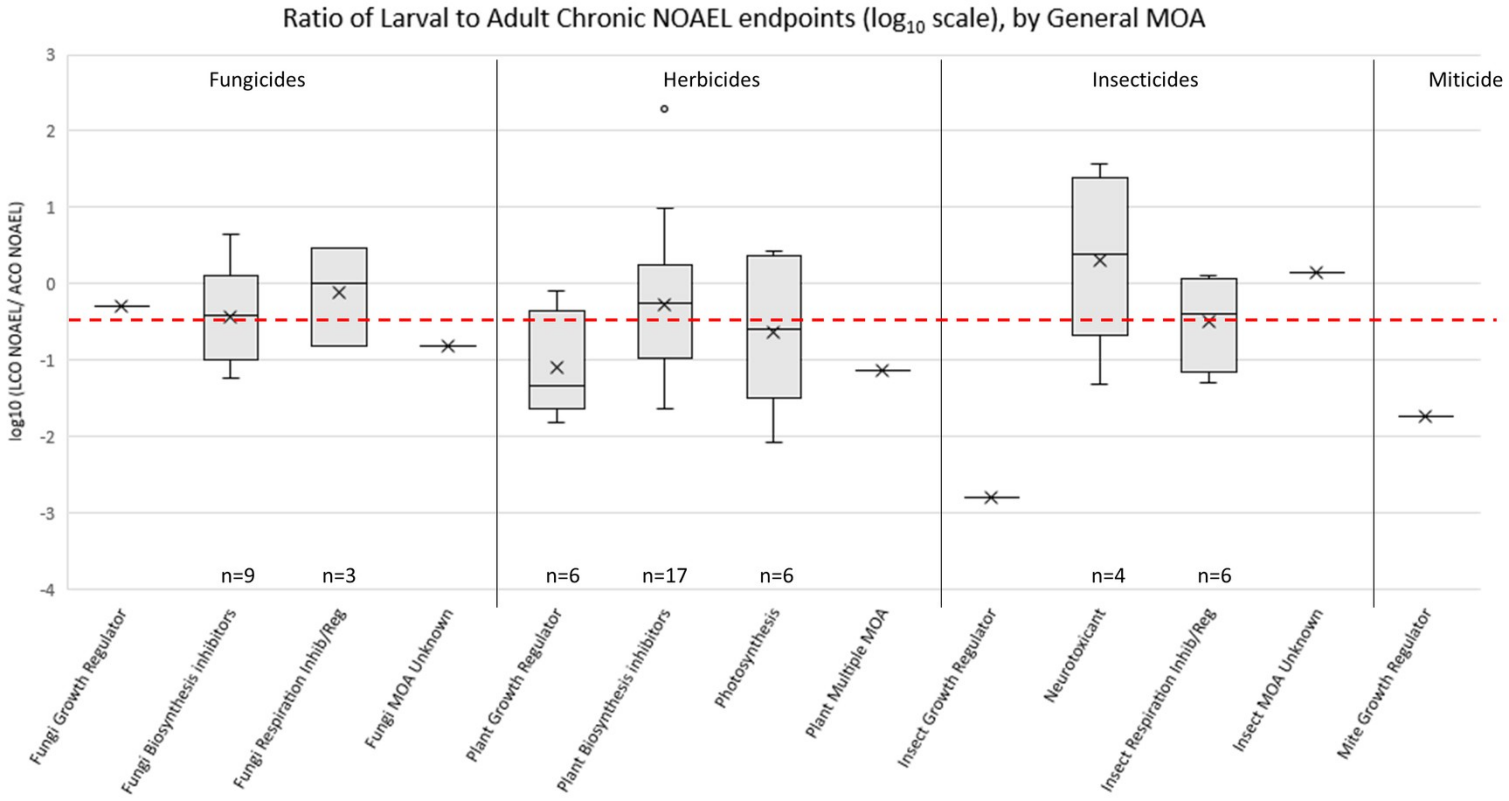
Ratio of honey bee (*Apis mellifera*) chronic mortality-based NOAEL endpoints from larval (LCO; OECD GD 239) and adult (ACO; OECD 245) toxicity studies. NOAEL ratios are organized by general mode of action (MOA), within pesticide type. Red dashed line represents the ratio of equivalence for dietary dose comparisons of larvae to adult bees. [sample sizes n>1 are provided below boxes, n of 1 is indicated with an “-x-“; shaded boxes represent first quartile to the third quartile, “x” represents the mean, horizontal bar is the median, whiskers extend out to the 95^th^ percentiles].

To look more closely at this pattern, comparisons of the NOAELs based on mortality were made between the ACO and LCO studies of 57 pesticides that had both studies. In this paired dataset, 28 of the pesticides did not report effects up to the highest concentration tested (*i*.*e*., non-definitive LOAELs) in the ACO study; whereas, for the LCO study, only 13 pesticides had non-definitive LOAEL values. For one pesticide (*i*.*e*., oxyfluorfen) there was no established NOAEL as the study had statistically significant effects at all test levels. The paired NOAEL endpoint comparisons span a variety of MOAs and chemical classes (Fig 5), but as a whole and across both chemical class and MOAs (other than for neurotoxic insecticides), the larval NOAEL values were generally more sensitive than their corresponding adult endpoints (log10 < 0), similar to the pattern observed across all available data (Fig 4). However, to understand this difference in terms of relative potential for risk, the data in Fig 5 are compared to the red dotted line in the figure. The red line represents the ratio under which a risk quotient would be equivalent based on default Bee-REX dietary dose exposure assumptions for larval and adult bees.

In contrast neurotoxic insecticides (n = 4) had a distribution of ratios that predominantly exceeded the red dashed line indicating a generally more sensitive adult NOAEL and a likely larger risk potential for adult bees. The larval NOAEL values were more sensitive than the adult NOAEL counterparts in for 40 (70%) of the 57 chemicals. Under the default exposure assumptions in risk assessment ([13]; based on differential growth stage and caste food consumption rates), the instances where the ratio is less than 0.42 indicates that the larval NOAELs are more sensitive than the adult NOAELs, and thus would result in a greater risk given the exposure assumptions. This occurs for 31 (54%) of the 57 chemicals (S1 Table).

### Acute-to-chronic ratio (ACR) analyses

**Fig 6:**
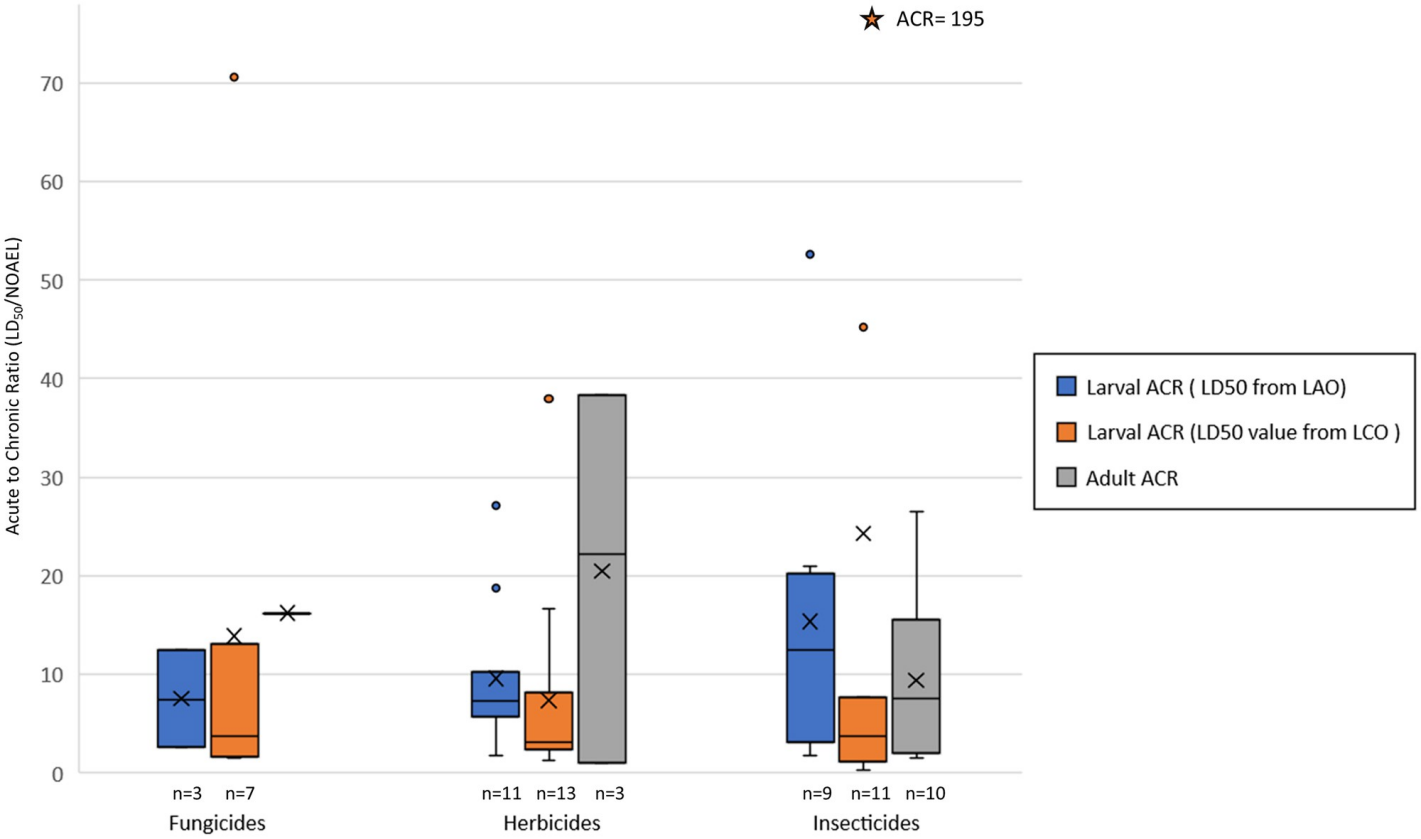
Larval and adult honey bee (*Apis mellifera*) acute-to-chronic ratios (ACRs), across pesticide types. The two types of larval ACRs are seperated, based on the source of the acute larval LD_50_ value (from either the LAO or LCO study). [sample sizes n>1 are provided below boxes, n of 1 is indicated with an”-x-“; shaded boxes represent first quartile to the third quartile, “x” represents the mean, horizontal bar is the median, whiskers extend out to the 95^th^ percentiles].

Acute-to-chronic ratios (ACRs) for adult honey bees were calculated using the AAO LD_50_ values and the ACO mortality-based NOAEL values. While there were 49 chemicals with both of these studies, only 14 had definitive values for both. In terms of the chemicals that had a non-definitive LD_50_ from the AAO study, 13 had reported mortality effects in their ACO studies and defined a LOAEL. For the 14 chemicals that had definitive values for both studies, the ACRs ranged from 0.94 to 38. The only pesticide with an ACR less than 1 was the herbicide bromoxynil, which had an ACR of 0.94. The other two herbicide adult ACRs were 22 and 38 (Fig 6).

The ACRs for larval honey bees were calculated using the LAO LD_50_ values relative to the most sensitive larval daily dose mortality or adult emergence-based NOAEL from the LCO study. Larval ACRs ranged from 1.8 to 52 for the 23 chemicals that had definitive acute LD_50_ values. There were another 17 chemicals with non-definitive LD_50_ values from the LAO study. Their LCO counterpart studies also did not define an LD_50_ based on 8-day mortality, and for 9 of them no effects were observed at the highest doses tested. An additional review of the larval ACR based on the LCO-derived LD_50_ allowed for 31 chemical comparisons (S5 Table). As indicated by the direct comparison of LD_50_ values from the LAO and LCO studies, the LCO study typically provided a more sensitive LD_50_ value than the LAO study; therefore, the ACRs for most chemicals are smaller (range from 0.23 to 195, Fig 6). Notably two ACRs were very different from the other estimates; the fungicide valifenalate (ACR = 70) and insecticide thiamethoxam (ACR = 195, indicated with the orange star in Fig 6). Both of these ACRs were generated from definitive LD_50_ values from LCO studies and had NOAECs that were bounded by LOAECs.

## Discussion

### Toxicity trends between oral and contact tests

Despite testing different exposure pathways, this retrospective analysis indicates that adult acute contact (AAC) and adult acute oral (AAO) LD_50_ endpoints exhibit a positive relationship (Spearman’s ρ = 0.74, p <0.0001, n = 49, Fig 2). These results indicate that, while differences in exposure pathway may influence the delivery of a chemical, the difference in tested pathway between AAO and AAC studies did not manifest in differing sensitivities towards a given compound in terms of LD_50_ values. The comparisons between the adult acute contact and oral LD_50_ values suggest that there were relatively similar toxicities reported for most chemicals between study types across pesticide classes, with oral toxicity being slightly more sensitive or as sensitive as the counterpart contact-based endpoint. Based on current assumptions used in USEPA’s pesticide risk assessment framework (See [13]), the difference between exposure to a pesticide through food consumption is modeled at higher rate (12x) relative to a contact exposure and as a result the contact toxicity of a compound would need to be 12x more toxic than the acute oral endpoint to elicit an equivalent level of risk. As indicated by the sensitivity ratio values (*R values*) between contact and oral endpoints, this analysis found that only 16% of chemicals had AAC LD_50_ values ≥12x AAO LD_50_ and the majority (84%) of compounds had less than a five-fold difference between LD_50_ values (Fig 3). This finding indicates that based on the data and exposure assumptions in USEPA’s risk assessment process, AAO LD_50_ values can be protective of AAC values. However, the value of using acute oral LD_50_ values as a surrogate for AAC studies may be limited when considering the loss of information provided by contact-specific endpoints. While the inclusion of a non-definitive/greater than (>) values introduces some uncertainty into the R values because the true endpoint value is unknown, it permits the inclusion of compounds with vastly different toxicities across exposure pathways (oral vs. contact) in the analysis. For example, half of the six pesticides had an *R value* ≥ 1 (indicating relatively higher contact toxicity) were in the pyrethroid insecticide class. This may indicate differences in the adsorption, distribution, metabolism, and/or excretion between contact and dietary exposure for this class of chemicals and may provide useful information when characterizing risks from these compounds. While two fungicides also had *R value* ≥ 1, they both had non-definitive acute oral LD_50_ values; therefore, caution should be exercised in interpreting the significance of this ratio for these pesticides. Ultimately, the inclusion of some non-definitive values results in more conservative ratios because the non-definitive endpoint value is an overestimation of toxicity.

### Acute toxicity to adult and larval honey bees

While the primary aim of larval acute oral toxicity studies (LAO) is to establish an LD_50_, larval chronic (repeat dose) oral toxicity studies (LCO) have also been used to derive an LD_50_ value that can be used as a surrogate for a single-dose LAO LD_50_. To account for the difference in exposure between the two study types, the comparison between the LAO and Day 8 LD_50_ from LCO studies values are made using a converted daily dose, where dosing is normalized to essentially an equivalent per day basis (μg/bee/day). We report that LD_50_ endpoints from LCO studies were more sensitive relative to LD_50_ values from LAO studies (Wilcoxon Signed Rank Test on matched pairs, n = 15 Z = -37, *p* = 0.03). The endpoints used in this analysis were all definitive. Despite being primarily used for determining chronic NOAEL values, LCO studies can yield LD_50_ values that are lower and protective of those calculated in LAO studies. It is important to consider that this comparison was limited to only fifteen pesticides, and that two of the chemicals resulted in more sensitive LD_50_ values generated from the LAO study. The inclusion of a greater number of pesticides and MOAs would strengthen the understanding of this relationship. However, based on our current dataset the LCO appears to generate LD_50_ endpoints which can be considered protective relative to the LAO LD_50_ values.

We also compared the distribution of acute toxicities for adults and larvae across pesticide types (*i*.*e*., insecticides, herbicides, fungicides, and other) using acute toxicity classifications (Fig 1). The overall distribution of pesticide types was similar between life stages (adults and larvae), but the proportion of toxicity values that were definitive was much greater for larvae than adults, and for insecticides as compared to other types of pesticides (especially in studies of adults). When comparing the number of definitive LD_50_ endpoints classified as “highly toxic”, the majority of these values were from studies testing insecticides (Fig 1). However, we report a greater number of herbicides and fungicides classified as moderately toxic to honey bee larvae as compared to adults, potentially because there were more definitive LD_50_ values in these studies. This highlights the importance of larval studies when characterizing the potential toxicities of pesticide types other than insecticides.

When examining differences between acute adult and larval toxicity studies, we only compared chemicals with definitive endpoints and only 11 pesticide active ingredients could be directly compared (Table 4). The analysis indicates that adult LD_50_ values were more sensitive than the larval LD_50_ values calculated in LAO studies (5 out of 8 studies). Conversely, larval chronic (LCO) LD_50_ values were generally more sensitive than the corresponding adult values in 88% of comparisons (7 out of 8 comparisons see Table 4). In ecological risk assessment, differences between larvae and adult diet composition and consumption rates impacts exposures of a pesticide to larvae and adults [13]. These difference in dietary dose-based exposures result in larval toxicity endpoints needing to be 2.36 times lower than the adult toxicity endpoints (ratio = 0.42) to result in the same level of risk [13]. Thus, the ratios less than 0.42 expressed in Table 4 indicate instances where the larval LD_50_ value would result in higher risk estimation based upon the default assumptions of exposure. Depending on the source of the larval LD_50_ (LAO or LCO), either 3/8 or 5/8 of the studies give a ratio below 0.42, indicating a greater risk for larval honey bees. However, the limited dataset will need to be expanded to determine if the larval honey bees are indeed more sensitive than the adults across broader pesticide and MOA groupings. This observation of greater larval sensitivity was also observed in the chronic toxicity studies, discussed below. We also report that thiamethoxam had the largest difference between adult and larval endpoints (see Table 4). This finding is in line with prior work demonstrating that adults are more sensitive to thiamethoxam [29]. No other neonicotinoid endpoints were available for this comparison, limiting our ability to draw conclusions about this effect based on insecticide class.

### Chronic toxicity to adult and larval honey bees

In order to examine differences in chronic toxicity between honey bee lifestages, adult chronic (ACO) and larval chronic oral (LCO) NOAEL values were compared. The NOAEL is defined as the highest tested dose level which does not yield a significant effect relative to the control group and consequently, both the biological response and selected test concentrations can influence this endpoint. A variety of considerations are made when selecting dose level in a chronic toxicity study such as the estimated environmental concentrations of a pesticide or the inclusion of a “limit dose.” The impact of these factors on test concentration selection undoubtedly influenced the endpoints included in our dataset such that many chemicals reported NOAELs without having a definitive LOAEL. For these reasons, selected dose-levels may not always identify the exposures at which a chronic effect may begin to occur but are useful when establishing exposures which are unlikely to elicit an effect in risk assessment. Ultimately, dosage-level selection may have impacted the interpretation of 12 of the 29 compared ACO and LCO pairs as one or both of the studies had reported no observed effects at the maximum doses tested. Given this assumption, the analysis indicates that larval chronic mortality NOAELs were generally more sensitive (lower) than the corresponding adult values (Fig 4). Research has shown that thermal, nutritional, and chemical stress during honey bee larval development can have latent impacts on the health of adult life stages [30–32]. The LCO study has been designed with the sensitivity of developmental stages in mind and is carried through to adult emergence (eclosion) to capture this potential increase in sensitivity across vulnerable developmental stages. Temporal trends among larval chronic toxicity data, where Day 22 NOAELs were more sensitive than Day 8 (larval) or Day 15 (pupal) NOAELs were observed across all pesticide types (Fig 4). As a result, the increased sensitivity observed at Day 22 may not always be detectable at earlier time points. Despite the cessation of larval feeding (and test substance exposure) just prior to pupation at Day 8, effects on survival during pupation and until adult emergence can be observed at dose levels far lower than those which elicited significant effects during earlier stages of development. This underscores the importance of continuing the chronic assay until eclosion as an adult bee.

Chronic mortality endpoints followed the same pattern as the acute toxicity studies regarding differences among types of pesticides. Insecticides, herbicides, and fungicides (Fig 4) were more toxic to larval bees than to adults. Insecticides have modes of action intended to cause adverse outcomes in insects. Not surprisingly, insecticides had the lowest NOAELs, with greater chronic toxicity for both adults and larvae relative to fungicides and herbicides. The chronic mortality endpoints can also be compared across larval and adult studies using the daily EEC ratio of 0.42, the point where despite differences in the default assumptions of exposure the toxicity endpoints would result in the same magnitude of risk. Roughly half of the pesticides had ratios below 0.42; 31 (54%) out of 57 chemicals had larval LD_50_ values more sensitive than the adult values. Therefore, in a risk assessment the likely result would be a greater estimation of risk for larvae (Fig 5, S1 Table). Four neurotoxic insecticides had a distribution of ratios that exceeded a ratio of 1 indicating a generally more sensitive adult chronic oral NOAEL (Fig 5), but the small sample size makes patterns across MOAs difficult to conclude. Our finding in this retrospective highlights the overall increased sensitivity of honey bee larvae relative to adults in response to acute exposure, but also indicated that the increased larval sensitivity may not translate to increased risk because of differential exposure potential related to the conservative assumptions that the risk assessment makes for daily consumption rates of adult (*i*.*e*., nectar foragers) versus larval honey bees.

### Acute-to-chronic (ACR) comparisons for adult and larval honey bees

The ACR is used in ecotoxicology to estimate chronic toxicity when well-defined (definitive) acute toxicity data exist (*i*.*e*., LD_50_ data) and the chronic data are unavailable ([28]; the acute toxicity endpoint may also be estimated from the chronic endpoint using an ACR). While this is not typically relied upon for honey bee toxicity evaluations (because of limited data), we conducted this analysis to provide insight of the acute to chronic toxicity relationships for honey bees. We compared ACRs for both adult and larval studies using the LD_50_ from the acute toxicity test guidelines and a defined NOAEL value from the chronic toxicity testing.

Larval ACRs were calculated for 23 and 31 pesticides for LAO and LCO-derived LD_50_ endpoints, respectively, and adult ACRs were calculated for 14 pesticides S5 Table and Fig 6). Larval ACRs ranged from 0.23–195 (average ACR = 13.4) based on either the LAO or LCO sourced LD_50_ values. This was similar to adult ACRs which ranged from 0.943 to 38.28 (average ACR = 12.3). In comparison to the NOAEL from the same study, the NOAEL was 195 times more sensitive than the repeat dose LD_50_. The adult ACR was 2, which also indicates a more sensitive endpoint in the chronic study, but the difference was far less extreme.

### Importance of life stage, study duration and pesticide risk evaluation

Insect pollinator testing is required to support risk assessment for the registration and re-registration of conventional pesticides in the United States (See FIFRA and 40CFR158). As a result, USEPA maintains one of the most comprehensive honey bee toxicity databases, containing toxicity endpoint data on both adult and larval life stages (ECOTOX- https://cfpub.epa.gov/ecotox/). Despite having a large honey bee toxicity database with relevant endpoints, the ability to generate direct comparisons across specific chemicals in this retrospective was limited by the extent of non-definitive values. Non-definitive values are useful for identifying dosages which are unlikely to elicit an effect for risk assessment purposes, but non-definitive values have limited utility in statistical analyses and direct comparisons across life stages. Another limitation of the available data for use in comparisons across tests and life stages is that resulting from studies that were conducted with a TEP as opposed to TGAI. The TEP studies represent approximately one third of all available adult contact and adult oral acute toxicity data. Spruill *et al*. [33] previously investigated the differences in relative toxicity classifications between TGAI and TEP, across both contact and oral exposure toxicity studies and found that the toxicity estimates for TEP data were similar to TGAI. A high-level comparison of the data available to USEPA also came to a similar conclusion. While the exclusion of studies and toxicity endpoints from the analyses presented here significantly reduced the dataset for comparisons, this ensured a more robust and scientifically defensible analysis.

The evaluation of data submitted subsequent to the release of USEPA’s *Guidance for Assessing Pesticide Risks to Bees* [13] and the development of the adult chronic oral, larval acute oral, and larval chronic oral test guidelines, illustrates the importance of these newer toxicity studies. These data demonstrate that there is a differential sensitivity to pesticides between adult and larval life stages. The data also show that both herbicides and fungicides are frequently identified as being toxic to larval bees and these toxicities are manifested more in the larval and chronic studies as compared to the acute adult studies. The data also show that the repeated dietary exposure to a pesticide can increase its toxicity to adult bees. This evaluation underscores the importance for pesticide studies on adult and larval honey bees despite their intended target pest (*e*.*g*., herbicides and fungicides), as well as the importance of having captured both acute and chronic responses to a pesticide exposure. Moreover, the relationships between study endpoints identified in these analyses may help inform future efforts to streamline pollinator toxicity data needs and reduce future animal testing.

## Supporting information

S1 Table Compendium of chemicals and available most sensitive endpoints for each guideline study after applying screening criteria described in the methods. References for each endpoint are provided in S4 Table.
(XLSX)

S2 Table Online sources of information related to mode of action (MOA) of pesticide to target species. (XLSX)

S3 Table Insecticide classifications with corresponding Insecticide Resistance Action Committee (IRAC) Mode of Action (MoA) classifications. (XLSX)

S4 Table Cross reference table and list of references (MRIDs) for each of the endpoints provided in S1 Table. (XLSX)

S5 Table Endpoints and calculated acute-to-chronic ratios (ACRs) for adult and larval honey bee (*Apis mellifera*) toxicity studies. (XLSX)
